# Flavonoid-modulated JAK-STAT signaling mitigates malignant transformation and drug resistance in breast tumors: A clinically relevant 3PM-guided innovation

**DOI:** 10.1016/j.jare.2025.10.067

**Published:** 2025-11-07

**Authors:** Peter Kubatka, Mykhailo Huniadi, Andrea Kapinova, Natalia Nosalova, Elizabeth Varghese, Dana Blahutova, Slavomir Hornak, Alexandra Trbolova, Kamil Biringer, Katarina Adamicova, Dasa Cizkova, Dietrich Büsselberg, Olga Golubnitschaja

**Affiliations:** a Center of Experimental and Clinical Regenerative Medicine, Small Animal Clinic, University of Veterinary Medicine and Pharmacy, 041 81 Kosice, Slovakia; b European Association for Predictive, Preventive and Personalised Medicine (EPMA), 1160 Brussels, Belgium; c Department of Biology and Ecology, Pedagogical Faculty, Catholic University in Ružomberok 034 01 Ružomberok, Slovakia; d Biomedical Centre Martin, Jessenius Faculty of Medicine, Comenius University in Bratislava, 036 01 Martin, Slovakia; e Department of Physiology and Biophysics, Weill Cornell Medicine in Qatar, Education City, Qatar Foundation, 24144 Doha, Qatar; f Small Animal Clinic, University of Veterinary Medicine and Pharmacy, 041 81 Kosice, Slovakia; g Clinic of Obsterics and Gynecology, Jessenius Faculty of Medicine, Comenius University in Bratislava, 036 01 Martin, Slovakia; h Department of Pathological Anatomy, Jessenius Faculty of Medicine, Comenius University in Bratislava, 036 01 Martin, Slovakia; i Institute of Neuroimmunology, Slovak Academy of Sciences, 845 10 Bratislava, Slovakia; j Predictive, Preventive and Personalised (3P) Medicine, Department of Radiation Oncology, University Hospital Bonn, Rheinische Friedrich-Wilhelms-Universität Bonn 53127 Bonn, Germany

**Keywords:** Breast carcinoma, Predictive preventive personalized medicine (PPPM / 3PM), Cancer chemo-resistance, Cell plasticity, Anti-cancer therapy, Re-sensitization, Flavonoids, JAK, STAT signaling, Multi-level diagnostics, Mitochondrial homeostasis and fitness, Artificial intelligence, Big data interpretation, Patient phenotyping and stratification, Treatments tailored to individualized patient profile, Primary and secondary care, Improved individual outcomes

## Abstract

**Background:**

Breast cancer (BC) treatment efficacy is often compromised by tumor cell plasticity and multidrug resistance of multi-factorial origin. Among emerging therapeutic agents, flavonoids − a structurally diverse group of naturally occurring polyphenols − have demonstrated a significant potential to modulate the Janus kinase/signal transducer and activator of transcription (JAK-STAT) signaling pathway, a key driver of oncogenic transformation, immune evasion, and therapeutic failure in BC. Notably, natural compounds, including but not restricted to apigenin, quercetin, naringenin, morin, luteolin, butein, xanthohumol, silibinin, baicalein, nobiletin, rosmanol, orientin, eriocitrin, breviscapine, 8-hydroxydaidzein, icariside I, cardamonin, epigallocatechin-3-gallate, fisetin, brutieridin, melitidin, isoliquiritigenin, and sophoraflavanone G, have been recognized as potent JAK-STAT modulators in BC models.

**Key scientific concepts of the study:**

Through targeted interference with this pathway, flavonoids exert pleiotropic antitumor effects − enhancing the efficacy of chemotherapeutic agents, suppressing cellular proliferation and invasion, and reducing tumor aggressiveness. Beyond JAK-STAT signaling, flavonoids also influence several additional therapy resistance-related mechanisms, including regulation of ATP-binding cassette (ABC) transporters, promotion of apoptosis, suppression of epithelial–mesenchymal transition (EMT) and cancer stem cells (CSCs), cell cycle arrest, mitochondrial autophagy and mitophagy modulation, as well as remodeling of the tumor microenvironment.

**Aims of the study:**

This article provides a comprehensive overview of the molecular mechanisms by which flavonoids overcome therapeutic resistance in BC, focusing on JAK-STAT signaling modulation. The paper follows principles of Predictive, Preventive, and Personalized Medicine (3PM), promoting the paradigm shift from reactive to proactive medicine. Contextually, it underscores the importance of multidisciplinary research to elucidate flavonoid-specific mechanisms, identify predictive biomarker panels, and develop advanced delivery systems, integrates patient phenotyping, multi-level diagnostics, and AI-driven data interpretation to enable cost-effective primary and secondary prevention, and therapeutic algorithms tailored to personalised patient profiles improving therefore individual outcomes.

## Highlights

• Persistent JAK/STAT activation drives resistance, metastasis, and immune evasion.
• Flavonoids (e.g., apigenin, quercetin) modulate JAK/STAT and resensitize.
• Flavonoids suppress EMT and stemness via STATs/microRNAs; limit plasticity, spread.
• Nanodelivery boosts flavonoid bioavailability and clinical effectiveness.
• 3PM plus AI enable personalized regimens and more cost-effective care.

## Graphical abstract

**Figure:**
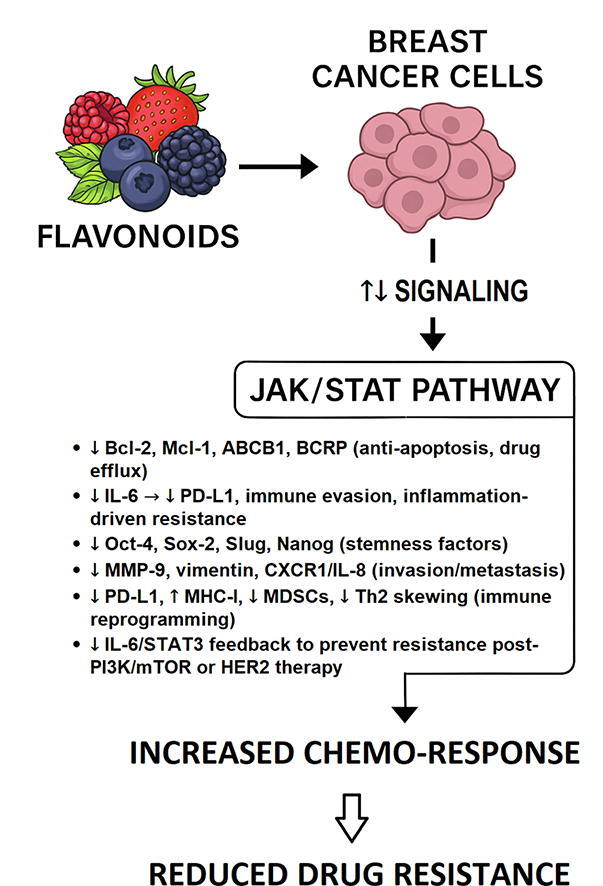

## Introduction

In oncology, signaling pathways and molecular networks are essential regulators of carcinogenesis, governing processes such as proliferation, apoptosis evasion, and cellular migration [1]. Receptors and ligands initiate upstream signaling, activating downstream cascades influenced by diverse intrinsic and extrinsic factors [2]. These complex networks contribute to cancer initiation, progression, and therapy resistance. Malignant transformation involves extensive alterations in signaling mechanisms, including genetic, transcriptomic, and epigenetic changes that converge on pathways controlling growth, survival, and motility. Disruptions also affect the tumor microenvironment (TME), angiogenesis, and inflammation, which collectively accelerate tumor progression [1,3].

Breast cancer (BC) is the most common heterogeneous malignancy and the second leading cause of cancer-related death in women [4]. Early detection through global screening has improved survival. While hereditary mutations in BRCA1/2, PTEN, and TP53 contribute to high-risk cases, receptor profiles (ER, PR, HER2) are pivotal for BC classification and pathogenesis [5,6].

A major challenge in BC therapy is acquired drug resistance, which emerges after initial treatment, diminishing therapeutic efficacy [7]. Resistance is driven by genetic alterations in tumor cells, changes in drug targets, and TME modifications. Despite advances, no universally effective treatment exists for metastatic or invasive BC. Resistance mechanisms include apoptosis inhibition, abnormal proliferation, EMT, autophagy, enhanced DNA repair, gene amplification, efflux transporter activation, and oncogenic pathway dysregulation (e.g., EGFR, PI3K/Akt, JAK-STAT, NF-κB) [[8], [9], [10], [11]]. Additionally, interactions between cancer cells and TME components − such as immune and stromal cells − contribute to resistance development. Drug resistance evolves dynamically, akin to a learning process. Cancer cells suppress inhibited pathways and strengthen compensatory ones, forming a “resistance memory” [12]. Key players in this adaptation include non-coding RNAs, scaffolding proteins, intrinsically disordered proteins (IDPs), protein translocation, and epigenetic modulators [13,14]. These elements also facilitate epithelial-mesenchymal transition (EMT), a key driver of metastasis and resistance. Importantly, phenotypic plasticity − central to EMT and resistance − is now considered a core hallmark of cancer [15].

To date, numerous canonical cancer-associated molecular pathways have been identified, such as the mitogen-activated protein kinase (MAPK) signaling cascade [16,17], the NF-κB pathway [18,19], the PI3K/Akt/mTOR axis [20,21], the Wnt/β-catenin signaling pathway [22,23], and the JAK-STAT signaling cascade [24]. The Janus kinase/signal transducer and activator of transcription (JAK/STAT) pathway is a central axis linking cytokine signaling to cellular outcomes such as proliferation, apoptosis, invasion, inflammation, tissue repair, and immune responses [25,26]. The JAK-STAT signaling cascade is an evolutionarily conserved pathway comprising ligand-receptor complexes, four JAK isoforms (JAK1, JAK2, JAK3, TYK2), and seven STAT proteins (STAT1, STAT2, STAT3, STAT4, STAT5a, STAT5b, and STAT6) [26]. Upon ligand binding, JAKs phosphorylate STATs, which dimerize, translocate to the nucleus, and regulate gene expression [27,28]. JAK-STAT signaling also interacts with other pathways to maintain cellular homeostasis and coordinate processes like hematopoiesis, immunity, and embryonic development [29].

Aberrant JAK-STAT activation drives oncogenesis, tumor progression, and therapy resistance by promoting uncontrolled proliferation, inhibiting apoptosis, and suppressing antitumor immunity [30]. Dysregulated signaling contributes to resistance to chemotherapy, targeted agents, and immunotherapies by activating survival pathways and altering drug responses. Emerging data suggest that combining JAK-STAT inhibitors with conventional therapies may overcome resistance and improve outcomes [[30], [31], [32]].

Current efforts aim to discover selective JAK-STAT modulators [33]. However, many exhibit toxicities or rapidly induce resistance. In contrast, plant-derived compounds such as flavonoids show promise in reversing multidrug resistance (MDR) in BC with minimal toxicity [34,35]. Combining conventional chemotherapeutics with flavonoids represents a promising strategy to enhance efficacy and counteract therapeutic resistance [36].

Flavonoids, a class of over 8000 plant-derived molecules, are widely distributed in foods, herbs, and medicinal plants [37]. These phytochemicals exert notable effects on the JAK-STAT signaling pathway, contributing to anti-inflammatory, antitumor, and cardioprotective activities [38]. As multi-target agents, flavonoids exhibit potent anticancer properties and can reverse tumor chemoresistance with low toxicity and high efficacy [17,18,39]. Specific flavonoids act as multitarget inhibitors by lowering cytokine or growth hormone levels that activate JAK/STAT proteins, thereby suppressing tumor cell proliferation. They modulate key oncogenic processes − cell proliferation, invasion, metastasis, immune evasion, angiogenesis, and apoptosis resistance − via JAK-STAT pathway inhibition [38,[40], [41], [42], [43], [44]]. In BC, flavonoids reduce JAK2 activity and inhibit STAT3 phosphorylation, downregulating STAT3-regulated targets such as VEGF, Bcl-2, Mcl-1, cyclin D, and survivin, while activating caspases-3, −7, and −9 to induce apoptosis and impair cell survival [41]. These findings underscore their potential as therapeutic or chemopreventive agents in BC management.

Despite increasing research, no comprehensive review has addressed how flavonoids modulate cancer cell plasticity in resistant BC through JAK-STAT signaling. This paper explores how flavonoids enhance therapy sensitivity by targeting this pathway. Following the 3PM framework, integrating flavonoids into combination regimens supports predictive diagnostics, early resistance detection, and personalized therapies. Future research should focus on identifying predictive biomarkers and molecular targets that sensitize resistant cells, prevent recurrence, and inhibit progenitor-like resistant phenotypes. When incorporated into individualized treatment regimens, flavonoids − combined with AI-driven diagnostics and health risk stratification − offer a promising, cost-effective approach aligned with 3PM principles and the shift from reactive to proactive medicine [36,[45], [46], [47], [48], [49]].

### Source of research data and criteria for Inclusion/Exclusion

Research data were collected from the PubMed database using keywords and MeSH terms such as “breast carcinoma,” “cell plasticity,” “resistance,” “JAK-STAT signaling,” and various flavonoid subclasses (e.g., “flavanones,” “flavonols,” “flavones,” “isoflavonoids,” “chalcones,” and “anthocyanidins”) in combination with “radiotherapy,” “chemotherapy,” “targeted therapy,” and other relevant terms.

**Inclusion criteria** for Heading 3 encompassed:

(a) studies on flavonoid effects in BC,
(b) administration of nanomaterials combined with flavonoids,
(c) flavonoid-associated modulation of JAK-STAT signaling,
(d) in vitro, animal, or human studies using natural or synthetic flavonoids,
(e) controlled experimental designs,
(f) studies using pure flavonoids or combination therapies,
(g) plant extract/herbal interventions containing flavonoids, and
(h) reports demonstrating modulation of cancer cell plasticity or reversal of drug resistance via JAK-STAT signaling.

**Exclusion criteria** for Heading 3 included:

(a) non-original research articles,
(b) interventions involving non-flavonoid phytochemicals or studies lacking precise dosing/duration data, and(c) studies focused on non-BC tumors or unrelated signaling pathways.

## Mechanisms of JAK-STAT pathway involvement in breast cancer resistance

### The JAK-STAT signaling pathway

The JAK-STAT signaling cascade is a pivotal intracellular network that transmits signals from cytokines, growth factors, and hormones to regulate gene expression. It orchestrates key cellular functions, including proliferation, differentiation, apoptosis, and immune responses [50]. The pathway consists of: (a) cytokine receptors that bind ligands such as interleukins, interferons, and growth factors; (b) Janus kinases (JAKs), which phosphorylate and activate downstream STAT proteins upon receptor stimulation; (c) signal transducers and activators of transcription (STATs), which dimerize after phosphorylation and translocate to the nucleus to regulate transcription; and (d) negative regulators, including SOCS, PIAS, and PTPs, that ensure pathway homeostasis and prevent aberrant activation [26,29,51].

JAK-STAT signaling encompasses several subpathways that mediate distinct physiological outcomes. The pathway is initiated by ligand-induced receptor dimerization and JAK activation, leading to STAT recruitment, phosphorylation, dimerization, and nuclear localization, ultimately driving expression of genes involved in inflammation, survival, and immunity [26,29]. Interleukin-driven pathways include IL-6/JAK-STAT3 (inflammation, cancer) [52]; IL-2/JAK1-JAK3/STAT5 (T-cell development) [53]; and IL-10/JAK1-TYK2/STAT3 (anti-inflammatory effects) [54]. Interferon signaling comprises: Type I IFNs (IFN-α/β)/JAK1-TYK2/STAT1-STAT2 (antiviral defense) [55]; Type II IFN (IFN-γ)/JAK1-JAK2/STAT1 (macrophage activation) [56]; and Type III IFN (IFN-λ)/JAK1-TYK2/STAT1-STAT2 (mucosal immunity) [57]. Hormonal subpathways include GH/JAK2/STAT5 (growth and metabolism) [58], EPO/JAK2/STAT5 (erythropoiesis) [59], TPO/JAK2/STAT5 (platelet production) [60], and leptin/JAK2/STAT3 (appetite and metabolic regulation) [61]. Collectively, the JAK-STAT system governs a wide array of biological processes, including immunity, hematopoiesis, and metabolic homeostasis, and its dysregulation is implicated in inflammatory diseases and cancers.

### The JAK/STAT signaling and cancer resistance

The JAK/STAT signaling pathway plays a central role in oncogenesis, tumor progression, and therapy resistance [31]. JAK kinases, constitutively associated with cytokine and hormone receptors, are rapidly activated upon ligand binding, initiating tyrosine phosphorylation cascades [62]. They are essential for interferon-mediated antitumor immunity [63], while aberrant activation contributes to malignant transformation and uncontrolled proliferation [64]. JAK1 facilitates IL-6 signaling, metastatic progression, and STAT3 activation in HER2-positive BC, promoting tumor growth and immune evasion [65]. JAK2 is a key driver of tumorigenesis, metastasis, and immunosuppression [66], whereas JAK3 is frequently active in lymphoid malignancies even in the absence of mutations [67]. TYK2, acting downstream of overactive growth factor receptors, is also implicated in tumorigenesis and may serve as a therapeutic target [68].

STAT proteins exhibit dual roles in cancer. While STAT3 and STAT5 are oncogenic, promoting tumor initiation and progression [[69], [70], [71]], STAT1 and STAT2 support antitumor immunity and immune surveillance [72]. Persistent JAK/STAT activation enhances tumor cell survival, proliferation, and invasiveness, making this axis a valuable target for therapeutic intervention [32]. Understanding the dynamic interaction between JAKs and STATs in solid tumors is essential for advancing targeted therapies [73].

JAK2/STAT3 signaling contributes significantly to chemoresistance. In nasopharyngeal carcinoma, this axis promotes paclitaxel (PTX) resistance via FOXM1 induction and anti-apoptotic gene expression [74,75]. JAK2 inhibitors can block P-glycoprotein (P-gp)-mediated drug efflux, reversing resistance in PTX-resistant cell lines [76]. Combining JAK/STAT inhibitors with conventional chemotherapy enhances efficacy. For instance, fedratinib sensitizes cells to vincristine by suppressing P-gp [77], ruxolitinib reverses chidamide resistance in NK/T-cell lymphoma by inhibiting STAT3 [78], and JAK2/PI3K co-inhibition counters IL-6-driven STAT3/STAT5-mediated resistance to PI3K inhibitors [77]. In EGFR-mutant NSCLC, EGFR activation triggers JAK1/STAT3 signaling, initially suppressed by EGFR-TKIs like erlotinib. Resistance mutations reduce TKI effectiveness, but JAK1 inhibitors (e.g., CJ14939) can restore drug sensitivity [78,79]. Zoledronic acid overcomes EMT-associated gefitinib resistance via JAK1/STAT3 inhibition [80]. In glioblastoma, STAT3 activation and MGMT overexpression limit temozolomide efficacy; resveratrol enhances response by inhibiting STAT3 and downregulating MGMT [81].

#### The role of JAK/STAT signaling in resistant breast carcinoma − therapeutic implications

Chemoresistance remains a major limitation in BC treatment, underscoring the need for novel strategies. Recent genomic and molecular studies have elucidated the critical role of JAK/STAT signaling in BC initiation, progression, and therapeutic resistance [82]. Deregulation of this pathway poses significant clinical challenges, with emerging data implicating dysregulated JAKs, STATs, and SOCS proteins in BC pathogenesis and drug resistance [32]. Notably, JAK2 and TYK2 have been associated with BC progression and resistance mechanisms [66,83]. STAT1 functions as either a tumor suppressor or an oncogene in BC [84], with high expression linked to therapeutic resistance and metastasis [85]. STAT3, frequently activated in primary BC, promotes invasion, metastasis, and poor prognosis and responsiveness to chemotherapeutics by modulating inflammatory responses and cytokine-driven tumor microenvironment interactions [86]. STAT3 activation promotes tumor growth and suppresses antitumor immunity via IL-10 [87]. Constitutive STAT5 signaling supports tumor cell survival, progression, metastasis, and resistance to anti-BC therapies [88]. STAT6, through IL-4–driven Th2 polarization, further facilitates immune evasion and tumor support [89,90].

**Fig. 1:**
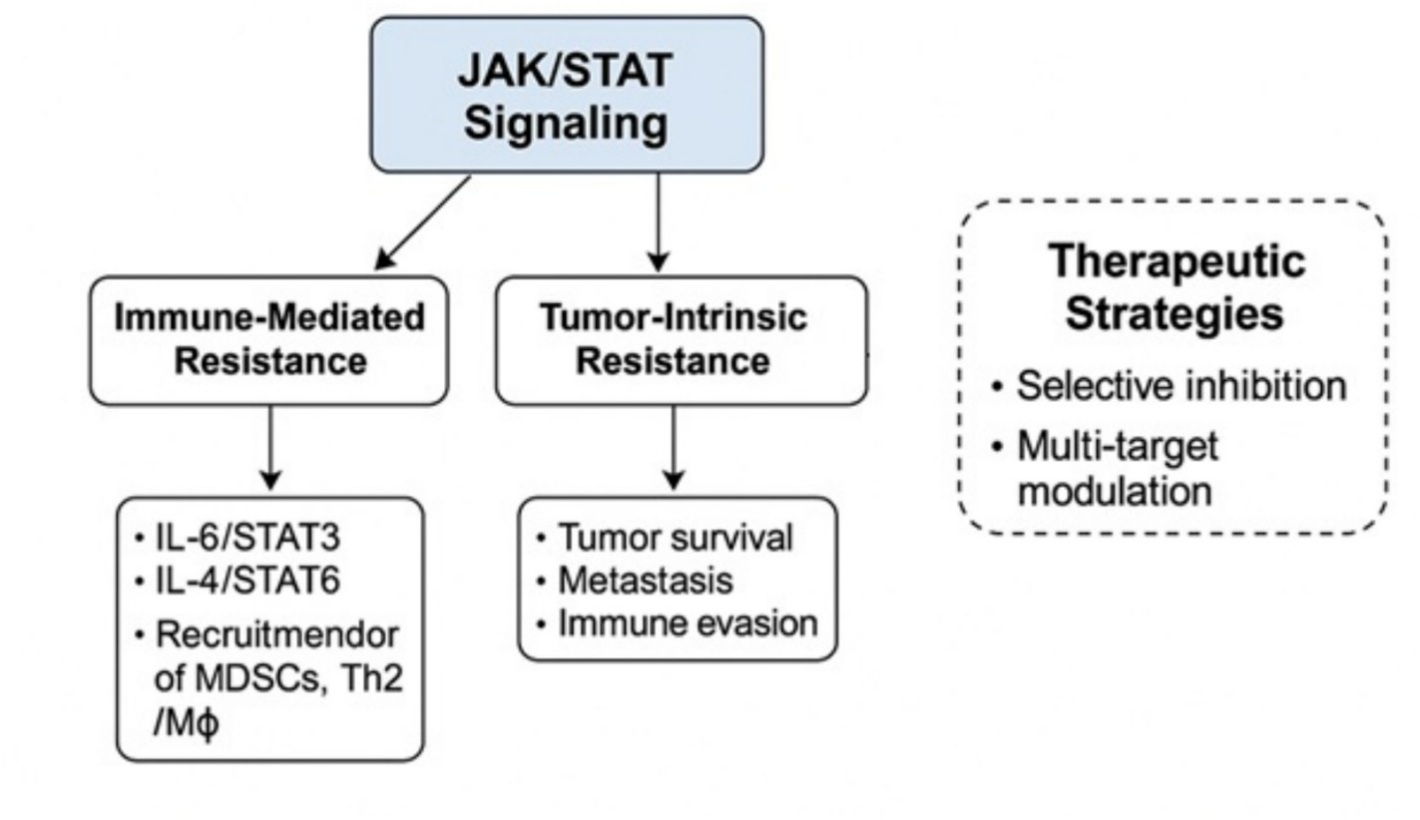
JAK/STAT Signaling in Resistant Breast Carcinoma.

Beyond tumor-intrinsic and stromal factors, immune evasion is a discrete, clinically relevant resistance mechanism directly wired to JAK/STAT. In breast tumors, STAT3 upregulates PD-L1 and dampens T-cell activity, linking oncogenic signaling to checkpoint escape [91,92]. Disruption of the interferon–JAK1/2–STAT1 axis (e.g., JAK1/2 loss-of-function) reduces MHC-I antigen presentation and PD-L1 inducibility, fostering primary or acquired resistance to PD-1/PD-L1 blockade [93]. In parallel, IL-6/STAT3 signaling expands myeloid-derived suppressor cells, establishing a tumor–MDSC feedback loop that promotes metastasis while suppressing immunity [94,95]. IL-4/STAT6 skews macrophages and T-helper cells toward Th2-biased, immunosuppressive states, with IL-4 cooperating with IL-6 to reinforce tumor-supportive phenotypes [96]. Taken together, checkpoint upregulation (PD-1/PD-L1), impaired antigen presentation (↓MHC-I), and recruitment/polarization of suppressive myeloid and Th2 programs comprise actionable JAK/STAT-linked resistance biology that must be addressed alongside tumor-intrinsic pathways in BC (Fig. 1) [92].

In biomarker-anchored BCs with clear JAK/STAT hyperactivation, selective inhibitors are preferred; they provide clean pharmacodynamic readouts, enable biomarker-guided enrollment, and minimize off-target burden. Yet early trials of JAK1/2 or STAT3 blockade in solid tumors, including BC, were largely tolerable but showed limited single-agent activity, consistent with pathway redundancy and rapid compensatory rewiring [[97], [98], [99], [100], [101]]. Where redundancy dominates, IL-6 → JAK/STAT3 feedback, stromal–myeloid crosstalk, and adaptive signaling loops, polypharmacologic (multi-target) strategies are better suited, concurrently dampening cooperating hubs and narrowing evolutionary escape [[102], [103], [104]]. In practice, selective inhibitors are prioritized for biomarker-defined, pathway-addicted disease, whereas multi-target modulation is reserved for resistance sustained by parallel circuits and tumor–microenvironment feedback. Selectivity is most appropriate when a single JAK/STAT node (e.g., JAK1/2 or STAT3) is the actionable driver and trials require clean PD/PK with biomarker stratification; however, data from BC studies with ruxolitinib combinations and early STAT3 inhibitors indicate that on-target suppression alone frequently fails to achieve durable control owing to network redundancy and rapid compensatory rewiring (Fig. 1) [[97], [98], [99],^105^].

#### Targetable JAK-STAT subpathways in chemoresistant breast cancer

**Fig. 2:**
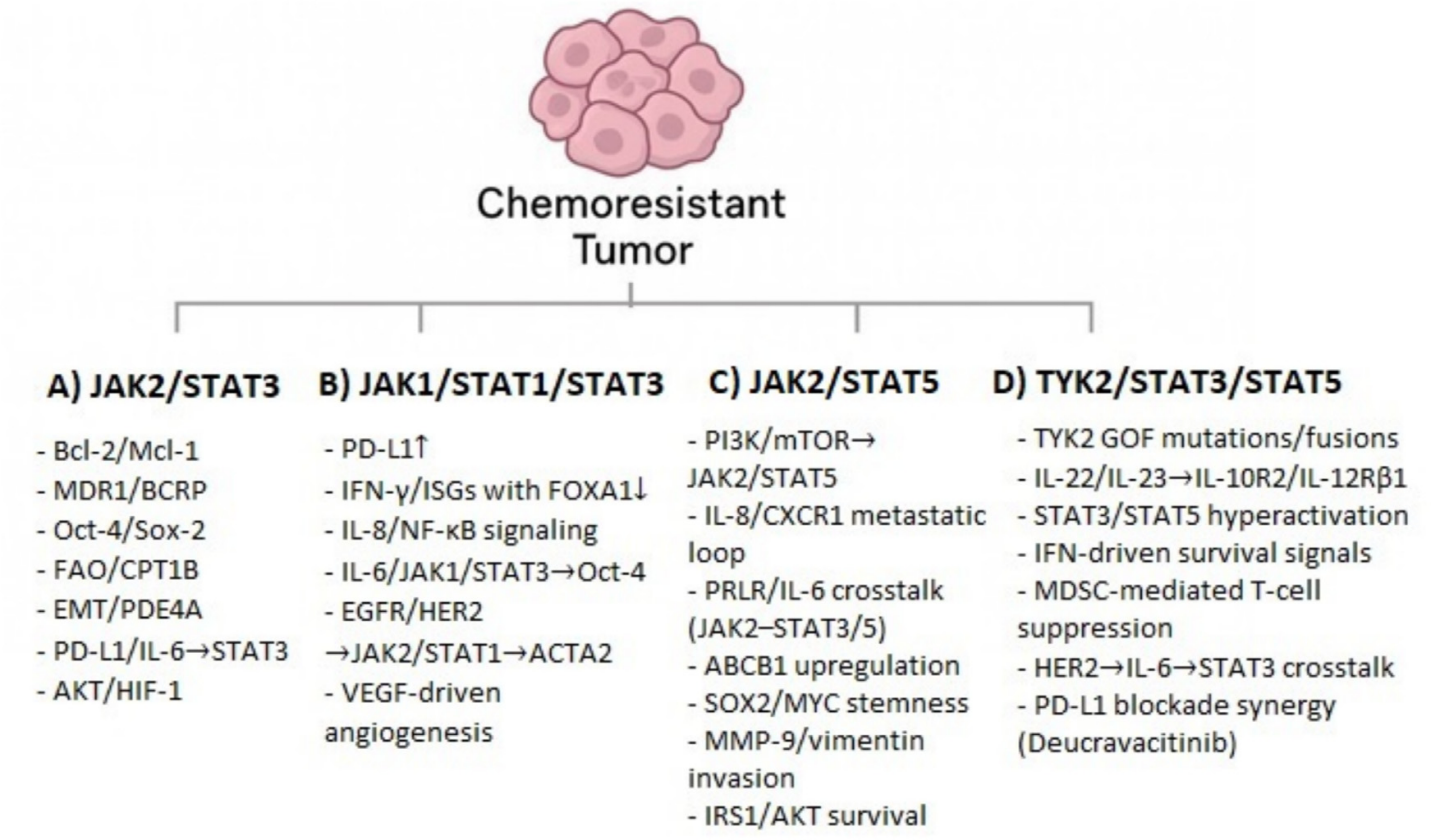
JAK/STAT subpathways driving chemoresistance in BC and their therapeutic implications.

Chemoresistant breast tumors frequently exhibit dysregulated JAK-STAT signaling, making it a crucial therapeutic target. The most relevant subpathways for modulation include (a) JAK2/STAT3 pathway, (b) JAK1/STAT1/STAT3 pathway, (c) JAK2/STAT5 pathway, and (d) TYK2/STAT3/STAT5 pathway (Fig. 2) [32,33,106].

*Targeting the JAK2/STAT3 Pathway*.

STAT3 plays a pivotal role in mammary gland development and is persistently activated in chemoresistant BC [107]. It drives resistance by upregulating anti-apoptotic proteins (Bcl-2, Mcl-1) and drug-efflux transporters (MDR1, BCRP) [108,109]. Additionally, JAK2/STAT3 signaling promotes proliferation, survival, invasion, metastasis, and immune evasion, while supporting BC stem cell (BCSC) properties like self-renewal and pluripotency [[110], [111], [112], [113]]. Chemoresistance is further linked to downstream mediators, including FAO, CPT1B, MAPK/Akt, HIF-1, and Oct-4, with JAK2/STAT3 enhancing FAO and CPT1B expression [114].

The IL-6/JAK/STAT3 axis is critical in BC progression [115]. Its dysregulation promotes inflammation, tumor growth, immune escape, and resistance to therapy [[116], [117], [118]]. In inflammatory BC (IBC), a highly aggressive subtype, pSTAT3 drives EMT and upregulates PDE4A, linking cAMP signaling to resistance [119]. The CD44^+^CD24^−^pSTAT3^+^ subpopulation, enriched in IBC, depends on JAK2/STAT3 signaling. Combining paclitaxel with JAK2/STAT3 inhibitors more effectively reduces xenograft tumor growth than either treatment alone [116,119].

In triple-negative BC (TNBC), JAK/STAT3 hyperactivation contributes to immune evasion, proliferation, metastasis, and therapy resistance [120]. Resistance to HSP90 inhibitors and paclitaxel correlates with IL-6/JAK2 signaling. JAK2 inhibitors (e.g., LY2784544, AZD1480, ruxolitinib) have shown efficacy in restoring drug sensitivity [121,122]. Alkaloid Piperlongumine (PL) also inhibits JAK2/STAT3, enhancing doxorubicin efficacy in TNBC [123].

STAT3 inhibitors like Stattic and Napabucasin demonstrate promising antitumor activity in preclinical models. Stattic inhibits IL-6-induced pSTAT3, induces apoptosis in TNBC cells, and targets BCSCs by downregulating stemness genes (Oct-4, Sox-2, Slug) [124,125]. It also potentiates doxorubicin by suppressing Bcl-2 and Bcl-xL [126]. Napabucasin, currently in clinical trials, targets CSCs and reduces CD44 expression via STAT3/STAT1 inhibition [118,127,128]. In cases of doxorubicin or capecitabine BC resistance, STAT3 inhibitors may provide a cost-effective alternative to monoclonal antibody therapies [118].

*Targeting the JAK1/STAT1/STAT3 Pathway*.

The JAK1/STAT1 axis plays a critical role in BC chemoresistance by promoting cell survival, proliferation, and resistance to apoptosis, thereby impairing chemotherapy efficacy [33]. While STAT1 is classically associated with interferon-induced tumor suppression, emerging evidence indicates its dual role: enhancing apoptosis in some contexts while contributing to therapy resistance and tumor progression in others [[129], [130], [131]]. Upon activation, STAT1 translocates to the nucleus and initiates transcription of interferon-stimulated genes (ISGs). IFN–γ–mediated JAK/STAT activation induces lineage plasticity in luminal bladder cancer by downregulating FOXA1 and upregulating basal markers and ISGs, with JAK1 inhibition reversing these changes [132]. Similarly, IFNγ/JAK1/STAT1/p65 NFκB signaling promotes IL–8–mediated tumor progression in ovarian cancer [133], while JAK1/STAT1 knockout impairs lineage plasticity in prostate cancer [134]. Overactivation of STAT1 is also associated with upregulation of PD-L1 and resistance to immunotherapy [135].

In chemoresistant MCF-7 cells, phosphorylation of STAT1(Y701), STAT3(Y705), and other STAT family members, along with JAK1 and JAK2 expression, was significantly elevated [136]. Standard ER-targeted therapy failed to suppress IL-6/STAT3-driven metastasis, whereas ruxolitinib effectively inhibited this pathway and reduced invasion in vivo, indicating IL-6/STAT3 activity independent of ER signaling [137]. Furthermore, IL-6/JAK1/STAT3/Oct-4 signaling promotes the transformation of non-stem cells into stem-like cells in BC [138]. In TNBC models, penta-O-galloyl-β-D-glucose suppressed tumor growth and metastasis by inhibiting JAK1/STAT3 [139]. EGFR/HER2 dimerization also promotes ACTA2 expression via JAK2/STAT1, enhancing invasiveness [140], while tannic acid regulates EGF-R/JAK2/STAT1/3 and P38/STAT1/p21^Waf1/Cip1 pathways, inducing G1 arrest and apoptosis [141].

A Phase I trial combining ruxolitinib with weekly paclitaxel in HER2-negative metastatic BC showed clinical activity and tolerability [97]. In tamoxifen-resistant MCF-7 cells, ruxolitinib reduced STAT3 phosphorylation, suppressed proliferation, migration, EMT, and VEGF-mediated angiogenesis, and significantly decreased tumor weight and vascularization in a chick embryo model [142]. Combined therapies involving PD-L1 inhibitors (e.g., atezolizumab) and JAK1 blockade show potential to counter immune evasion and survival signaling in both early and triple-negative BC, offering a synergistic therapeutic strategy [143,144].

*Targeting the JAK2/STAT5 Pathway*.

JAK2 and STAT5 regulate the proliferation, differentiation, and survival of mammary epithelial cells. Aberrant JAK2-STAT5 signaling is implicated in developmental abnormalities and pathologies, including BC [145]. IL-6-mediated activation of STAT5 and STAT3 plays a critical role in therapeutic resistance [77]. Notably, JAK2-STAT5 and JAK2-STAT3, acting downstream of IL-6R and PRLR, may synergistically drive luminal BC progression, underscoring their relevance in ER-positive tumors [146].

Britschgi et al. demonstrated that JAK2/STAT5 hyperactivation upon PI3K/mTOR inhibition enhances TNBC invasiveness and metastasis by restoring Akt activity and upregulating IL-8. In early resistance, insulin receptor/IRS1 signaling activates JAK2/STAT5 and PI3K/Akt; subsequently, STAT5-induced IL-8 binds CXCR1, further amplifying metastatic signaling [147,148]. CXCR1, predominantly expressed in circulating tumor cells, is sensitive to JAK2 inhibition. Dual inhibition of PI3K/mTOR and JAK2/STAT5 targets CXCR1^+^ tumor-initiating cells, offering a strategy to suppress metastasis and resistance [149].

An in vitro study evaluated co-targeting JAK2 and TrkA in HER2-positive and TNBC cells. The Pacritinib-Entrectinib combination reduced BC stemness markers (SOX2, MYC), induced apoptosis, and suppressed orthotopic growth of trastuzumab-refractory HER2^+^ xenografts and basal-like PDXs. It also decreased brain and bone metastases without toxicity [150].

Li et al. showed that STAT5a and ABCB1 are co-upregulated in doxorubicin-resistant BC cells and chemoresistant patients. Pimozide, a STAT5 inhibitor, enhanced doxorubicin sensitivity by suppressing STAT5a activity and ABCB1 expression in vitro and in vivo [151]. Additionally, pimozide was effective against tamoxifen- and radiation-resistant BC models [152]. Dees et al. demonstrated its ability to inhibit invasion and migration across TNBC subtypes via suppression of STAT3 (Tyr705), MMP-9, and vimentin expression [153].

*Targeting the TYK2/STAT3/STAT5 Pathway*.

Multi-omics analyses have identified tyrosine kinase 2 (TYK2) as a frequently altered oncogene across various cancers and metastatic contexts [154]. As a member of the JAK family, TYK2 mediates cytokine-driven immune and inflammatory responses, while its aberrant activation fosters cancer cell proliferation, invasion, and resistance to apoptosis [155]. TYK2 primarily exerts oncogenic effects through activation of STAT3/STAT5 signaling, notably downstream of IL-22 and IL-23 (via IL-10R2 and IL-12Rβ1), which regulate tumorigenesis, metastasis, and inflammation [156,157]. STAT3 also modulates interferon (IFN)-induced survival signals [70,158,159], and although TYK2-STAT5 signaling is less characterized, it may contribute to oncogenesis in specific contexts [160]. TYK2 gain-of-function mutations or gene fusions amplify STAT1/3/5 activation, driving tumor progression, immune evasion, and chemoresistance in BC [[161], [162], [163], [164], [165]].

Zhang et al. demonstrated TYK2′s tumor-suppressive role in BC, showing that Tyk2−/− mice injected with 4 T1 cells had accelerated tumor growth. This was not due to altered CD4^+^, CD8^+^, or NK cell function but rather enhanced suppression of T-cell responses by myeloid-derived suppressor cells [83]. Another study investigated Deucravacitinib, a selective TYK2 inhibitor, combined with the PD-L1 blocker INCB086550 in two TNBC models (syngeneic 4 T1/hPD-L1 and PBMC-humanized MDA-MB-231). TYK2 inhibition enhanced the antitumor efficacy of PD-L1 blockade with good tolerability [166].

Though TYK2′s direct involvement in HER2 signaling remains unclear, its role in activating STAT3/5 suggests an indirect contribution to HER2^+^ BC progression and trastuzumab resistance. HER2 overexpression induces IL-6, activating STAT3 and contributing to therapy resistance [111,167,168]. Trastuzumab’s efficacy may be compromised by alternative pathway activation (e.g., PI3K/Akt, MAPK) [[169], [170], [171]]. Thus, co-targeting TYK2/STAT3/STAT5 alongside EGFR/HER2 inhibitors (e.g., lapatinib, trastuzumab) could enhance treatment outcomes, though clinical validation is pending.

SAR-20347 is a selective TYK2/JAK inhibitor (IC_50_: 0.6 nM for TYK2, 23–41 nM for JAKs) [172]. In HT-29 cells, it potently inhibited IL-22-induced pSTAT3 (IC_50_: 148 nM) and achieved 94 % inhibition at 10 μM, comparable to effects in MSD assays for IL-12/pSTAT4 and IL-6/pSTAT3 [173]. In preclinical T-ALL models, SAR-20347 reduced STAT phosphorylation and IFN production, yielding tumor regression [174]. Further investigation is warranted to assess its utility in resistant BC.

The JAK2/STAT3 and JAK1/STAT1/STAT3 pathways play central roles in chemoresistant BC, especially in triple-negative and endocrine-resistant subtypes [119,120,149,[175], [176], [177]]. Additionally, JAK2/STAT5 and TYK2/STAT3/STAT5 signaling contribute to therapy resistance in BC (Fig. 2) [65,161,162,178]. Targeting these axes using synthetic JAK/STAT inhibitors, immunotherapeutics, or phytochemical-based agents holds promise for restoring drug sensitivity and enhancing treatment efficacy.

## Flavonoids as regulators of JAK-STAT signaling and their influence on breast cancer cell plasticity

**Fig. 3:**
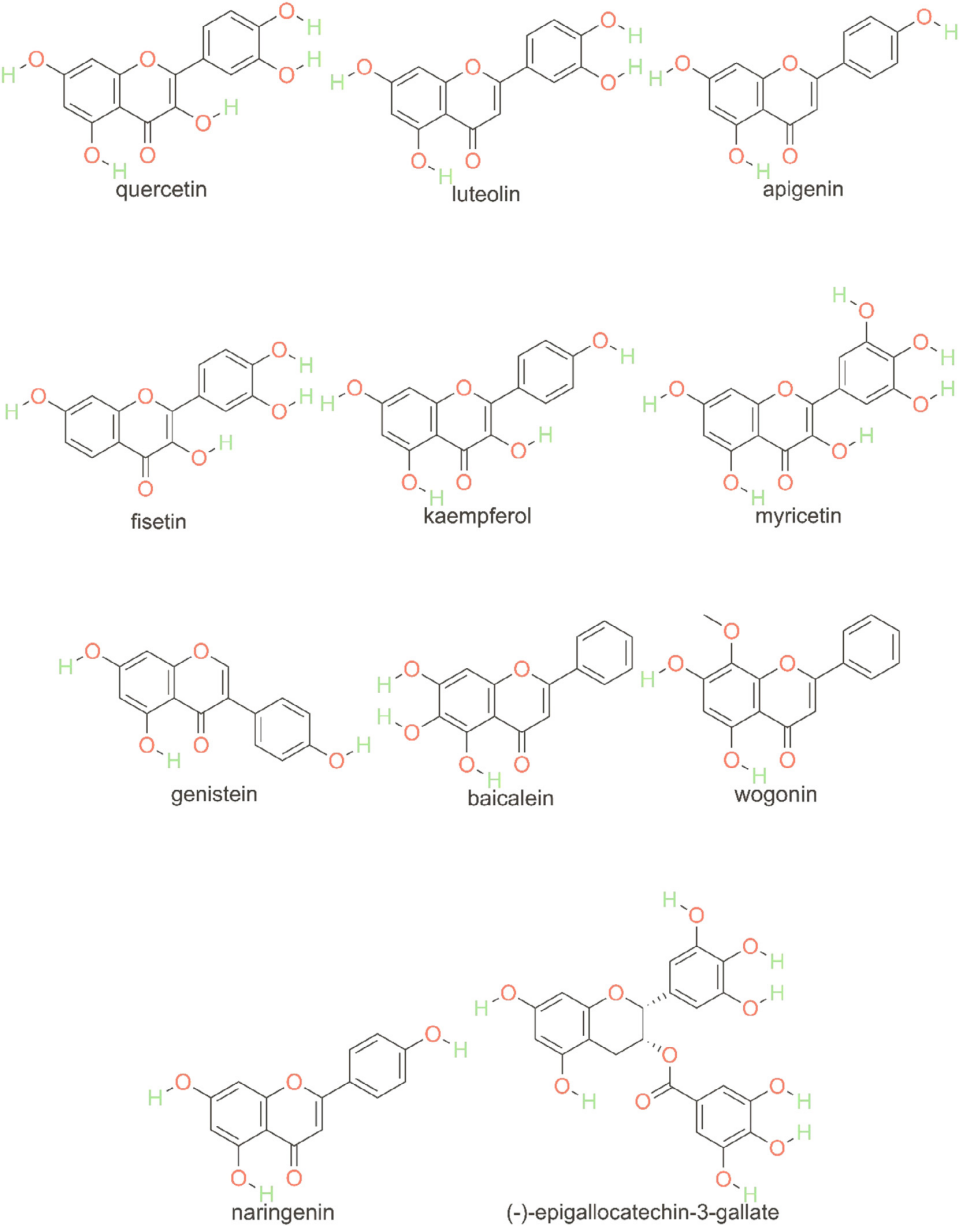
Representative flavonoid scaffolds with relevance to breast cancer and JAK/STAT modulation.

The JAK-STAT pathway plays a central role in chemoresistance, survival, and metastasis of aggressive cancers, making it a critical therapeutic target. Although JAK-STAT inhibitors are clinically available, their efficacy is often limited by resistance and adverse effects [[179], [180], [181]]. In contrast, flavonoids offer a promising alternative due to their low toxicity, potent activity, and accessibility [182,183]. Diet-derived flavonoids typically act as modulators, producing partial, context-dependent tuning across JAK/STAT–NF-κB–PI3K/AKT–MAPK axes rather than full blockade, while undergoing rapid phase-II conjugation (UGT/SULT), yielding broad safety margins seen across human and translational studies [[184], [185], [186]]. By modulating JAK-STAT signaling, flavonoids significantly impact breast carcinogenesis [26]. Dysregulated subpathways − JAK2/STAT3, JAK1/STAT1/STAT3, JAK2/STAT5, and TYK2/STAT3/STAT5 − promote proliferation, apoptosis resistance, and immune evasion in BC [38,187]. Flavonoids can counteract these mechanisms, enhancing chemo- and radiosensitivity, reducing toxicity, and improving therapeutic efficacy [39,188,189]. Representative flavonoid scaffolds with relevance to BC and JAK/STAT modulation are depicted in Fig. 3.

Despite these advantages, several factors hinder clinical translation beyond poor bioavailability. It consists of: (i) source/lot variability and extraction-driven shifts in aglycone–glycoside profiles, requiring batch-linked fingerprinting [190]; (ii) polypharmacology that reduces selectivity and introduces off-target/PK liabilities via UGT/SULT and efflux transporters (P-gp, BCRP, MRPs), necessitating prospective DDI assessment [191]; (iii) formulation constraints − poor solubility, instability, first-pass conjugation, variable permeability − that depress exposure unless resolved with defined delivery systems [192]; and (iv) inter-individual variation in microbiome and host metabolism (including enterohepatic recycling and deconjugation) that widens exposure distributions and confounds dose–response and biomarker anchoring [193].

### Flavonoids and JAK2/STAT3 pathway

Apigenin is one of the most extensively studied flavonoids, particularly for its modulation of the JAK2/STAT3 pathway in BC. In MCF-7 and doxorubicin-resistant MCF-7R cells, apigenin significantly reduced cell viability and reversed drug resistance by downregulating MDR1 expression at both mRNA and protein levels. It also suppressed JAK2 and STAT3 phosphorylation in both cell lines [44]. In HER2-expressing BT-474 cells, apigenin inhibited proliferation and induced caspase-dependent extrinsic apoptosis, associated with reduced p-JAK1, p-JAK2, and p-STAT3 expression and STAT3 nuclear translocation. It also blocked CoCl_2_-induced VEGF secretion and STAT3-dependent transcriptional activity [194]. Similarly, in HER2-overexpressing MDA-MB-453 cells, apigenin promoted PARP cleavage, induced apoptosis, and inhibited STAT3 signaling and VEGF release [195]. In SKBR3 cells, apigenin suppressed proliferation and induced caspase-dependent apoptosis, reduced p-JAK2/STAT3 levels, and blocked STAT3 nuclear localization and CoCl_2_-induced VEGF secretion − effects confirmed by the STAT3 inhibitor S31-201 [196]. In MCF-7/ADR cells, apigenin inhibited proliferation, colony formation, and reversed adriamycin resistance by downregulating MDR1, MRP, and P-glycoprotein expression and increasing rhodamine 123 accumulation. These effects were attributed to STAT3 inhibition and reduced secretion of VEGF and MMP-9 [197]. In vivo, apigenin treatment significantly suppressed tumor growth in MDA-MB-231 xenografts and downregulated pSTAT3, pERK, IL-6, PI3K, pAkt, and N-cadherin levels [198].

Quercetin is a natural flavonoid with strong antioxidant and anticancer properties. In MDA-MB-231 BCE cells, its combination with docetaxel (7 nM and 95 μM, respectively) significantly reduced cell viability and induced apoptosis with a synergistic effect (combination index = 0.76). This was linked to upregulation of p53 and BAX, and downregulation of BCL2, pERK1/2, Akt, and STAT3, indicating apoptosis induction and JAK2/STAT3 pathway inhibition [199]. Another study demonstrated that quercetin suppresses the proliferation, migration, and invasion of 4 T1 cells while promoting apoptosis and reducing IL-6 secretion. This was accompanied by decreased p-JAK2/JAK2 and p-STAT3/STAT3 expression, suggesting disruption of IL-6/JAK2/STAT3 signaling. Quercetin also reduced Treg cells, suppressed IL-10, and increased TNF-α, improving the tumor immune microenvironment [42]. Furthermore, folic acid–modified liposomal quercetin (FLQ) inhibited MDA-MB-231 cell growth by collapsing mitochondrial membrane potential and increasing ROS. FLQ downregulated JAK2/STAT3 phosphorylation and antiapoptotic proteins (Bcl2, Bcl-xL), while upregulating Bax, Bak, cytochrome *C*, and cleaved caspase-3, supporting a mitochondrial apoptosis mechanism [200].

Several studies have evaluated chalcones − a flavonoid subclass − for their modulation of JAK2/STAT3 signaling in BC. Butein, a chalcone, suppresses MDA-MB-231 cell viability, colony formation, and tumor growth in vivo without observable toxicity, while also reducing cell migration and invasion. These effects are primarily mediated by STAT3 inhibition, PARP cleavage, and apoptosis induction. When combined with frondoside-A, butein further enhances apoptosis and suppresses migration in MDA-MB-231 and A549 cells [201]. Isoliquiritigenin (ISL), derived from licorice, exerts anti-BC activity by suppressing STAT3 and its downstream target miR-21. It also upregulates PIAS3, a STAT3 inhibitor, and siRNA-mediated PIAS3 knockdown abolishes ISL’s inhibitory effect, confirming PIAS3′s essential role [202]. Xanthohumol sensitizes adriamycin-resistant MCF-7 cells to radiation, enhancing apoptosis by downregulating MDR1, EGFR, and STAT3, and upregulating DR4 and DR5 [203]. A synthesized chalcone-syringaldehyde hybrid (CSH1) demonstrated anti-TNBC effects by targeting CKAP2-mediated FAK and STAT3 phosphorylation, highlighting its therapeutic promise [204]. Additionally, the isoliquiritigenin analog 2′,4′,4-trihydroxychalcone (TEC) reduced BT549 cell proliferation, migration, and tumor growth, while inducing S-phase arrest. Transcriptomic analysis identified its mechanism as JAK2/STAT3 and p53 pathway inhibition, promoting a senescence-associated secretory phenotype (SASP) [205].

Studies combining flavonoids with standard chemotherapeutics highlight their efficacy in modulating JAK2/STAT3 signaling in BC. Naringenin enhanced cyclophosphamide's anticancer effects in MDA-MB-231 cells by inhibiting JAK2/STAT3 signaling, increasing BAX, reducing Bcl-2, and activating caspases 3 and 9. It also suppressed IL-6-regulated apoptosis genes [206]. In a murine mammary tumor model, naringenin with cryptotanshinone reprogrammed immune responses toward a Th1 phenotype by inhibiting IL-4 and increasing IFN-γ, while reducing intratumoral CD4^+^CD25^+^Foxp3^+^ T cells via JAK2/STAT3 modulation, indicating immunotherapeutic potential [207].

Co-treatment with morin and doxorubicin synergistically inhibited TNBC cell growth by increasing drug uptake, DNA damage, and activation of PARP and caspase-7. It also downregulated EGFR/STAT3 phosphorylation, FOXM1, RAD51, and survivin, and induced G2/M and S phase arrest, suggesting enhanced efficacy through EGFR/STAT3 pathway suppression [208]. Similarly, luteolin combined with paclitaxel increased apoptosis in MDA-MB-231 cells, as confirmed by DAPI and Annexin-V assays. The combination activated caspase-8 and −3, elevated Fas expression, and inhibited STAT3, significantly reducing tumor volume in xenograft models [209].

Several studies have confirmed the efficacy of flavonoids in modulating the JAK2/STAT3 pathway in BC. Silibinin (200 µM) inhibited MDA-MB-231 proliferation by suppressing JAK2/STAT3 phosphorylation, blocking STAT3 nuclear translocation, and downregulating MMP2. STAT3 knockdown reduced invasion, confirming its role in metastasis and MMP2 regulation [210]. Baicalein suppressed IL-6 production and STAT3 activation, reducing viability and migration of 4 T1 and MDA-MB-231 cells (IC50 = 10–100 µM), and inhibited metastasis in a 4 T1 mouse model [211].

Nobiletin inhibited angiogenesis in ER^+^ MCF-7 and T47D cells via Src/FAK/STAT3 signaling modulation through Paxillin [212]. A (+)-catechin-lysine complex (Cat:Lys) selectively inhibited viability and migration in BC, pancreatic, and colorectal cancer cells, acting through JAK2/STAT3 and Wnt pathway suppression [213].

8-Hydroxydaidzein (8-OHD) downregulated stemness markers, induced intrinsic apoptosis, and inhibited IL-6-induced JAK2/STAT3 phosphorylation in MCF-7 cells [214]. Rosmanol selectively inhibited proliferation and promoted apoptosis in MCF-7 and MDA-MB-231 cells, linked to S-phase arrest, ROS generation, mitochondrial disruption, and JAK2/STAT3 and ERK pathway inhibition [215].

Orientin reduced MCF-7 migration and invasion by downregulating MMP-9 and IL-8 through inhibition of TPA-induced PKCα, ERK, AP-1, and STAT3 nuclear translocation [216]. Icariside I suppressed proliferation and metastasis in 4 T1-bearing mice by targeting IL-6/JAK/STAT3 signaling [217].

Breviscapine modulated JAK2/STAT3 in a dose-dependent manner, regulating MCF-7 viability, cell cycle, and apoptosis [218]. Eriocitrin inhibited JAK2 and Src kinases, suppressing STAT3 phosphorylation and nuclear translocation, while inducing apoptosis through ROS generation and JNK/p38 MAPK activation [219].

Finally, current findings highlight the potential of flavonoid-rich plant extracts in modulating JAK2/STAT3 signaling in BC. Radix Glycyrrhiza (RG), known for its anti-inflammatory and antitumor effects, exerts its action partly through total flavonoids (TFRG), which modulate ERK/NF-κB/miR-155 signaling and downregulate iNOS in LPS/IFN-γ-stimulated RAW264.7 macrophages without cytotoxicity. TFRG significantly suppressed tumor growth in MDA-MB-231 xenografts, accompanied by reduced iNOS, 3-nitrotyrosine (3-NT) formation, and JAK2/STAT3 inhibition [220]. Aryappalli et al. showed that Manuka honey flavonoids suppress p-STAT3 activation in MDA-MB-231 cells by antagonizing IL-6R. ELISA assays revealed that four major flavonoids (luteolin, quercetin, galangin, chrysin) inhibited IL-6 binding to IL-6Rα (30–35 % at 50 μM) and downregulated p-STAT3 in a dose-dependent manner (IC50: 3.5–70 μM). Molecular docking confirmed IL-6Rα binding at sites disrupting ligand interaction, and p-STAT3 suppression was linked with reduced gp130 and p-JAK2 levels [221]. Rosa sterilis juice (RSJ), another flavonoid-rich extract, inhibited proliferation, clonogenicity, and induced mitochondrial apoptosis in BC cells. In 4 T1 xenografts, RSJ reduced tumor volume, histopathological lesions, Ki67 expression, and modulated apoptotic proteins via inhibition of JAK2/STAT3 signaling [222].

These findings highlight the ability of diverse flavonoids to modulate critical signaling pathways, especially JAK2/STAT3, in controlling BC progression and cellular plasticity for effective disease management (Table 1).

**Table 1:** Overview of flavonoid effects on BC cells by modulating JAK2/STAT3 upstream and downstream signaling pathways.

**Flavonoid**	**Study details**	**Affected process**	**Mechanism**	**Ref.**
Apigenin	MCF-7 and MCF-7R cells	Reduction of the viability of both cell lines	↓ MDR1, p-JAK2, p-STAT3	[44]
Apigenin	HER2-expressing BT-474 cells	Inhibition of BT-474 cell proliferation and induction of extrinsic apoptosis	↓ p-JAK1, p-JAK2, and p-STAT3	[194]
			↑ caspase-8, caspase-3, and PARP cleavage	
Apigenin	HER2-overexpressing MDA-MB-453 cells	Induction of extrinsic apoptosis	↓ JAK2/STAT3 phosphorylation, ↓ STAT3 nuclear localization, and ↓VEGF secretion	[195]
Apigenin	SKBR3 cells	Decreased cell proliferation and promoted apoptosis	↓ JAK2/STAT3 phosphorylation, ↓ STAT3 nuclear localization, and ↓VEGF secretion	[196]
Apigenin	MCF-7 and MCF-7/ADR cells	Decreased cell growth, colony formation, and induced apoptosis	↓ p-STAT3, VEGF, and MMP-9	[197]
Apigenin	MDA-MB-231 xenograft model	Decreased tumor growth	↓ p-STAT3, p-ERK, IL-6, PI3K, p-Akt, N-cadherin	[198]
Quercetin	MDA-MB-231 cells	Decreased cell viability, induced apoptosis, and increased sensitivity to docetaxel	↓ STAT3, BCL2, pERK1/2, Akt, ↑ p53 and BAX	[199]
Quercetin	4 T1 cells	Decreased proliferation, migration, and invasion, and promotion of apoptosis	↓ p-JAK2/JAK2 and p-STAT3/STAT3	[42]
			↓ IL-6/JAK2/STAT3 signaling	
Folic acid–modified liposome quercetin	MDA-MB-231 cells	Inhibited proliferation and promoted apoptosis	↓ JAK2 and STAT3 phosphorylation, mitochondrial membrane potential, Bcl2, Bcl-xL	[200]
			↑ ROS, Bax, Bak, cytochrome *C*, Cleaved-Caspase-3	
Butein	MDA-MB-231 cell	Suppressed viability, colony formation, tumor growth in vivo, and invasiveness, and induced cell death	↓ STAT3 inhibition,	[201]
			↑ PARP cleavage	
Isoliquiritigenin	Hs-578 T and MDA-MB-231 cells	Suppression of cell invasiveness	↓ PIAS3/STAT3/miR-21 signaling axis	[202]
Xanthohumol	Adriamycin-resistant MCF-7 cells	Apoptosis-driven enhancement of radiation response	↓ STAT3, MDR1, EGFR, Bcl-2, Bcl-x/L, and survivin	[203]
			↑ DR4 and DR5	
Chalcone-syringaldehyde hybrid	MCF-7, MDA-MB-231, MDA-MB-453 cells	Inhibition of DNA replication, cell cycle arrest, apoptosis, and decreased cell migration	↓ CKAP2-mediated FAK and STAT3 phosphorylation,	[204]
			↑ DNA damage	
2′,4′,4-trihydroxychalcone	BT549 cells	Suppressed proliferation, induction of cell cycle arrest, decreased migration in vitro, and reduced tumor growth in vivo, senescence-associated secretory phenotype	↓ JAK2/STAT3 and P53 pathways	[205]
Naringenin	MDA-MB-231 cells	Potentiated cyclophosphamide's anticancer effects	↓ JAK2/STAT3 signaling, IL-6, Bcl-2,	[206]
			↑ BAX, caspases 3 and 9	
Naringenin	Spontaneous mouse mammary tumor model	Decreased tumor volume, immunomodulatory effects, and synergism in combination with cryptotanshinone	↓ JAK2/STAT3 pathway, IL-4, intra-tumoral CD4^+^CD25^+^Foxp3^+^ T cell populations	[207]
			↑ IFN-γ secretion	
Morin	MDA-MB-231 cells	Inhibition of cell growth by enhancing doxorubicin uptake	↑ DNA damage, cleaved PARP and caspase-7, G2/M and S phase arrest,	[208]
			↓ EGFR/STAT3 phosphorylation, FOXM1, RAD51, survivin	
Luteolin	MDA-MB-231 cells and xenograft model	Anti-proliferative effects and apoptosis, synergistic effects with paclitaxel, and suppressed tumor volume	↓ STAT3,	[209]
			↑ caspase-8, caspase-3, Fas	
Silibinin	MDA-MB-231 cells	Suppressed proliferation and invasion	↓ JAK2/STAT3 phosphorylation, STAT3 nuclear translocation, MMP2	[210]
Baicalein	4 T1 and MDA-MB-231 cells	Suppressed cell viability and migration	↓ IL-6 production and STAT3	[211]
Nobiletin	MCF-7 and T47D cells	Inhibition of angiogenesis	↓ Src/FAK/STAT3 signaling via Paxillin	[212]
			↓ EGFR activity	
(+)-catechin-lysine complex	MCF-7 and MDA-MB-231 cells	Inhibition of proliferation, viability, and migration	↓ JAK2/STAT3 and Wnt pathway	[213]
8-Hydroxydaidzein	MCF-7 cells	Anti-stemness activity and apoptosis via the intrinsic pathway.	↓ IL-6-induced JAK2/STAT3 phosphorylation	[214]
Rosmanol	MCF-7 and MDA-MB-231 cells	Inhibited proliferation, cell cycle arrest, and induction of apoptosis	↓ ERK and JAK2/STAT3 ↑ ROS production, and DNA damage	[215]
Orientin	MCF-7 cells	Suppressed migration and invasion	↓ MMP-9 and IL-8 expression,	[216]
			↓TPA-induced PKCα and ERK activation,	
			↓ nuclear translocation of AP-1 and STAT3	
Icariside I	4 T1 in vitro and mouse model	Inhibition of lung metastasis of BC, suppression of BC proliferation, and induction of apoptosis	↓ IL-6/JAK/STAT3 signaling pathway	[217]
Breviscapine	MCF-7 cells	Regulation of cell viability, cycle arrest, and apoptosis		[218]
Eriocitrin	MCF-7 cells	Disrupting mitochondrial membrane potential and inducing apoptosis	↑ ROS levels, JNK/p38 MAPK	[219]
			↓ STAT3 phosphorylation and nuclear translocation	
			↓ JAK2 and Src kinase	
Radix Glycyrrhiza	MDA-MB-231 BCE xenografts	Suppression of tumor growth, anti-inflammatory effects	↓ JAK2/STAT3 pathway,	[220]
			↓ ERK/NF-*κ*B/miR-155 signaling, iNOS expression, 3-nitrotyrosine formation	
Manuka honey flavonoids	MDA-MB-231 cells	IL-6 receptor antagonism of Manuka Honey flavonoids	↓ p-JAK2, p-STAT3	[221]
Rosa sterilis juice	in vitro and 4 T1 xenograft models	Suppression of proliferation, altering morphology, decreased clonogenic potential, inducing apoptosis in vitro, and inducing apoptosis, inhibiting tumor growth, and mitigating pathological lesions in vivo	↓ Jak2/Stat3 signaling pathway in vivo and in vitro	[222]
			↓ Bcl-2 and Pro-caspase 3, Ki67	
			↑ P53 and Bax	

↑ upregulation; ↓ downregulation

### Flavonoids and JAK1/STAT1/STAT3 pathway

Combining flavonoids with chemotherapeutics represents a promising strategy for enhancing anticancer efficacy. Heba et al. (2024) showed that naringin synergistically enhanced doxorubicin's effects in MCF-7 cells by downregulating JAK1/STAT3 signaling. This combination reduced Bcl-2, Survivin, and VEGF levels while increasing Bax expression, thereby inhibiting proliferation and migration [223]. Similarly, apigenin suppressed HER2-overexpressing BT-474 cells by reducing JAK1, JAK2, and STAT3 phosphorylation, inhibiting STAT3-dependent transcription, and triggering extrinsic apoptosis through caspase-8 activation and PARP cleavage, without involving the intrinsic mitochondrial pathway [194]. The same group further demonstrated apigenin’s inhibition of STAT3 signaling in MCF-7 and HER2-overexpressing MCF-7 cells, along with reduced HER2 phosphorylation and increased p53, p-p53, and p21 expression, supporting its potential for treating HER2-positive BC [224]. Moreover, apigenin modulates immune checkpoints by suppressing IFN-γ-induced PD-L1 expression in multiple BC cell lines (MDA-MB-468, SK-BR-3, 4 T1) and human mammary epithelial cells, while its metabolite luteolin shows similar effects via reduced STAT1 phosphorylation (Tyr701, Ser727). Apigenin also enhanced IL-2 production and proliferation in PD-1^+^ Jurkat T cells co-cultured with MDA-MB-468 cells, indicating improved immune-mediated cytotoxicity [225].

Quercetin exhibits significant anticancer activity in BC models through modulation of the JAK/STAT signaling pathway and induction of apoptosis. In BC cell lines (MCF-7, MCF-10AT, MCF-10A, MDA-MB-231), quercetin triggers apoptosis via regulation of IFNγ-R, p-JAK2, and p-STAT1, while promoting γδ T cell differentiation, thereby enhancing immune-mediated tumor suppression [226]. In HER2-overexpressing BT-474 cells, quercetin inhibits proliferation and clonogenic survival in a dose- and time-dependent manner. It induces caspase-dependent extrinsic apoptosis (cleaved caspase-8, caspase-3, PARP) without involving mitochondrial pathways. Moreover, quercetin suppresses JAK1/STAT3 phosphorylation, STAT3 nuclear translocation, transcriptional activity, and MMP-9 secretion, collectively impeding tumor progression [227].

Cardamonin, a natural chalcone, modulates the immune microenvironment in TNBC by targeting JAK/STAT signaling. In MDA-MB-231 cells, it downregulates PD-L1, JAK1, and STAT3, while in MDA-MB-468 cells, it reduces STAT3 but increases JAK1. It also upregulates Nrf2, suppresses MUC1 and NF-κB1/2, and reduces CCL2 secretion, resulting in decreased macrophage recruitment and enhanced T cell infiltration, potentially sensitizing tumors to PD-1/PD-L1 inhibitors [228].

Flavonoid-rich lemon water extract, containing hesperidin, eriocitrin, and naringenin, exhibits antiproliferative effects in MDA-MB-231 cells (IC_50_ = 48.67 % at 1:32 dilution). This correlates with upregulation of JAK1, JAK2, TYK2, IRF3, and IRF7, indicating JAK/STAT pathway activation [229].

Green tea catechins, especially epigallocatechin gallate (EGCG), modulate JAK/STAT1 signaling through multiple mechanisms. EGCG more effectively inhibits STAT1 activity in endocrine-resistant MCF-7/LCC1 and MCF-7/LCC9 cells than in parental MCF-7 cells. Its combination with tamoxifen exhibits additive effects, highlighting potential in overcoming endocrine therapy resistance [178]. In MDA-MB-231 cells, 15 synthetic catechins structurally similar to EGCG were assessed for anti-STAT1 activity. The presence of three hydroxyl groups on the B ring and one on the D ring was essential. Surface plasmon resonance and molecular modeling identified two STAT1 binding sites, with site b showing higher affinity. Mutational analysis confirmed Gln518 and His568 as critical residues. Catechins also suppressed JAK2-mediated STAT1 phosphorylation, suggesting direct STAT1 inhibition [230]. Polyphenon E (Poly E), a standardized green tea extract, inhibits migration and reduces VEGF and MMP-9 expression in MDA-MB-231 and human endothelial cells. It also suppresses VEGF-induced neovascularization in vivo. Mechanistically, Poly E blocks STAT signaling by inhibiting IFN-γ-induced transcription and STAT1/STAT3 dimerization. Notably, constitutively active STAT3 attenuates Poly E's inhibitory effects, indicating its role in angiogenesis and BC progression via STAT1/3 inhibition [231].

Nobiletin, a citrus-derived polymethoxylated flavonoid, exhibits anti-metastatic effects in BC by modulating key signaling cascades. It inhibits migration and invasion by downregulating ERK/STAT and JNK/c-JUN pathways, thereby reducing transcription factors including STAT1, STAT3, ATF2, JUN, FOS, and ELK1 [232].

Brutieridin and melitidin (BM), flavonoids found in bergamot, exhibit statin-like effects by inhibiting HMG-CoA reductase in the mevalonate pathway. BM suppresses CSC traits by decreasing aldehyde dehydrogenase activity, mammosphere formation, and modulating STAT1/3, Notch, and Wnt/β-catenin pathways [233].

Fisetin, despite its known anticancer activity, is limited by poor solubility. Fisetin-loaded micelles enhance bioavailability and radiosensitizing efficacy in colorectal (CT26) and breast (4 T1) cancer cells, sparing normal fibroblasts (L929). In murine models, these micelles amplified radiotherapy efficacy without toxicity, attributed to inhibition of PDGFRβ/STAT1/STAT3/Bcl-2 signaling [234].

Co-treatment with luteolin and curcumin significantly enhanced STAT1 phosphorylation and OAS1 expression in MDA-MB-231 cells and xenografts compared to curcumin alone, indicating synergistic activation of the interferon/TGF-β/STAT1 axis, effectively suppressing triple-negative BC [235].

Collectively, these studies highlight flavonoids as modulators of JAK1/STAT1/STAT3 signaling, underscoring their therapeutic potential in BC management and cellular plasticity regulation (Table 2).

**Table 2:** Overview of flavonoid effects on BC cells by modulating JAK1/STAT1 upstream and downstream signaling pathways.

Flavonoid	Study details	Affected process	Mechanism	Ref.
Naringin	MCF-7 cells treated with the naringin + doxorubicin combination	Decreased proliferation and migration	↓ JAK1, STAT3	[223]
			↓ Bcl-2, Bax, VEGF, Survivin	
Apigenin	Effect on HER2^+^ BT-474 cell line	Inhibition of clonogenic survival of BT-474	↓ p-JAK1/2, p-STAT3	[194]
			↑ caspase-3, −8, PARP	
	Effect on HER2^+^ MCF-7, vector MCF-7	Induced apoptosis (extrinsic pathway), reduced phosphorylation of key proteins	↓ p-JAK1, p-STAT3	[224]
			↑ p53, p21, caspase-8, PARP	
	MDA-MB-468, HER2^+^ SK-BR-3, 4 T1 mammary carcinomas	Anti-tumor immune responses	↓ p-STAT1	[225]
			↓ PD-L1	
			↑ IL-2	
Quercetin	MCF-7, MCF-10AT, MCF-10A, MDA-MB-231 cell lines	Cell death, boosting the differentiation of γδ T cells	↓ p-JAK2, and p-STAT1	[226]
	HER2^+^ BT-474 cell line	Inhibition of proliferation, induction of apoptosis	↓ p-JAK1, p-STAT3, MMP-9	[227]
			↑ caspase-3, −8, PARP	
Cardamonin	MDA-MB-231 (MDA-231), MDA-MB-468 (MDA-468) cell lines	Promotion of T cell infiltration	↓ JAK1, STAT3, MUC1, NF-κB1, and NF-κB2, PD-L1 (MDA-231)	[228]
			↑ JAK1 (MDA-468)	
			↑ Nrf2 (both cell lines)	
			↓ NF-κB1 (MDA-468)	
Lemon extract	MDA-MB-231 cells	Inhibition of proliferation	↑ JAK1, JAK2, TYK2, IRF3, and IRF7 gene expressions	[229]
EGCG	endocrine-resistant MCF-7/LCC1 and MCF-7/LCC9	Influence of JAK1/STAT1 signaling	↓ JAK1, STAT1	[178]
		inhibition of STAT1		
	MDA-MB-231 cell line	Inhibition of STAT1	↓ p-JAK2, p-STAT1	[230]
Pol E	MDA-MB231 cell line	Reduced migration, antiangiogenic effect	↓ VEGF, MMP-9	[231]
			↓STAT3, STAT1	
Nobiletin	BC cells	Inhibition of migration and invasion	↓ STAT1, STAT3, ATF2, JUN, FOS and ELK1 mRNA	[232]
Brutieridin and Melitidin	CSC, CAF in TNBC	Inhibition of …	↓ STAT1, STAT3, Notch, and Wnt/β-catenin, ALDH, HMGR	[233]
Fisetin	CT26 and 4 T1 pathological cell lines, L929 normal cell line	Increasing the radiosensitivity of tumors	↓ PDGFRβ, STAT1, STAT3, Bcl-2	[234]
Luteolin	MDA-MB-231 and xenograft mice	Curcumin-induced synergistic therapy	↑ IFN1, ↓ TGF-β,	[235]
			↑ STAT1 and OAS1	

↑ upregulation; ↓ downregulation

### Flavonoids and JAK2/STAT5 pathway

Isoliquiritigenin (ISL), a natural chalcone found in various plants, possesses notable anticancer, antioxidant, and anti-inflammatory activities [236]. It inhibits BC cell proliferation, migration, and invasion by modulating oncogenic pathways [237]. However, its clinical translation is limited by poor solubility and stability. To overcome these challenges, ISL-loaded zein phosphatidylcholine nanoparticles (ISL@ZLH NPs) were developed [238]. Ganesan et al. demonstrated that ISL@ZLH NPs suppress BC-induced osteoclastogenesis and metastasis by downregulating STAT3, STAT5, and p-STAT5 in RAW264.7 cells treated with MDA-MB-231 conditioned medium. These results suggest the potential of ISL@ZLH NPs in preventing BC-associated bone metastases via JAK-STAT pathway inhibition [239].

Sophoraflavanone G, a flavonoid derived from Sophora species, is known for its diverse bioactivities, including anti-inflammatory and antimicrobial effects [[240], [241], [242]]. In MDA-MB-468 BCE cells, it inhibited STAT5b transcriptional activity, suppressed STAT protein phosphorylation, and downregulated upstream regulators JAK1 and JAK2. These effects correlated with apoptosis induction and reduced cell proliferation, highlighting Sophoraflavanone G as a promising small-molecule inhibitor of the JAK/STAT pathway for anticancer therapy [242].

Hwanggeumchal sorghum extract (HSE) contains diverse polyphenols, including anthocyanidins, phenolic acids, tannins, and phytosterols − bioactive compounds with antioxidant, anticancer, and hypolipidemic effects [243]. Park et al. (2012) demonstrated that HSE significantly suppressed tumor growth and metastasis in a BC xenograft model by inducing G1 phase arrest and downregulating oncogenic proteins. The anti-metastatic effects were attributed to inhibition of the JAK/STAT pathway, with reduced phosphorylation of STAT3, STAT5, and JAK2 in MDA-MB-231, MCF-7, and SKBR-3 cells. Furthermore, HSE exerted anti-angiogenic effects by downregulating VEGF and VEGF-R2 expression. Both in vitro and in vivo results confirmed that HSE limits BC progression through JAK/STAT pathway modulation [244].

Similarly, Oleaga et al. (2012) reported that coffee-derived polyphenols, rich in flavanols, catechins, flavones, and flavanones [245], modulate STAT5b expression in MCF-7 BC cells [246]. While STAT5b levels increased following treatment, cyclin D1, its downstream effector, was significantly downregulated. These findings suggest that coffee polyphenols may influence tumor signaling in a multifaceted manner and could be explored as dietary adjuncts in BC management [246].

JAK2 and STAT5a signaling has been shown to suppress early invasion and metastatic potential in BC cell lines (e.g., MDA-MB-231, MDA-MB-468, MDA-MB-157) by inhibiting EMT and promoting differentiation. A recent study reported that mistletoe (Viscum album L.) extract, rich in flavonoids, combined with garlic-derived diallyl trisulfide, upregulated JAK2 and STAT5a in BC cells. This activation triggered caspase-3-mediated apoptosis and significantly reduced cell invasiveness and metastatic capacity, suggesting therapeutic potential via pro-apoptotic and anti-metastatic mechanisms [247].

Genistein, a soy isoflavone, directly inhibits STAT5 phosphorylation in lactating mammary epithelial cells [248]. As STAT5 regulates genes involved in proliferation and survival, its inhibition may underlie genistein’s anticancer activity, which is also linked to modulation of the MEK5/ERK5/NF-κB pathway [249].

Collectively, these findings highlight JAK2/STAT5 as a promising therapeutic target for flavonoid-based interventions in BC (Table 3).

**Table 3:** Summary of flavonoid impacts on BC cells via modulation of upstream and downstream JAK2/STAT5 signaling pathways.

Flavonoid	Study details	Affected process	Mechanism	Ref.
Isoliquiritigenin-zein phosphatidylcholine nanoparticles	RAW264.7 cells treated with MDA-MB-231 conditioned medium in vitro	Inhibition of BC-induced bone damage and metastasis	↓ STAT3	[239]
			↓ STAT5	
			↓ p-STAT5	
Sophoraflavanone G	MDA-MB-468 cells in vitro	Apoptosis induction, suppression of proliferation	↓ STAT5b phosphorylation	[242]
			↓ JAK1, JAK2	
Hwanggeumchal sorghum extracts	MDA-MB-231, MCF-7 and SKBR-3 in vitro and xenograft models	Induction of cell cycle arrest, suppression of oncogenic proteins, reduction of tumor growth and metastasis, suppression of angiogenesis	↓ JAK2	[244]
			↓ STAT3 phosphorylation	
			↓ STAT5 phosphorylation	
			↓ VEGF, VEGF-R2	
Coffee polyphenols	MCF-7 cells in vitro	Reducing the risk of cancer, coadjuvant treatment	↑ STAT5b	[246]
			↓ cyclin D1	
Mistletoe extract	MDA-MB-231, MDA-468 and MDA-157 cells in vitro	Suppression of early invasion and metastasis, and apoptosis induction	↑ expression of JAK2	[247]
			↑ expression of STAT5a	
			↑ activation of caspase-3	

↑ upregulation; ↓ downregulation

### Flavonoids and TYK2/STAT pathway

Lemons contain several bioactive flavonoids, including eriocitrin, narirutin, hesperidin, neohesperidin, and neoeriocitrin [250]. In MDA-MB-231 triple-negative BC cells, Citrus limon water extract at 1:32 dilution exhibited an IC_50_ of 48.67 % in a neutral red uptake assay. Mechanistically, this extract significantly upregulated key components of the JAK/STAT pathway − JAK1, JAK2, TYK2 − and downstream interferon regulatory factors IRF3 and IRF7 [229]. IRF7, a central transcriptional activator of type I interferons, plays a tumor-suppressive role by limiting growth and metastasis [251], while aberrant JAK/STAT signaling, particularly via STAT3, is associated with BC progression and therapy resistance.

Although direct evidence of TYK2 modulation by flavonoids in BC is limited, several studies support their role in regulating JAK/STAT signaling. The flavone cirsiliol directly binds and inhibits TYK2, suppressing STAT3 activation in carcinoma models​ [252]. Quercetin blocks IL-12-induced phosphorylation of JAK2, TYK2, STAT3, and STAT4 in activated T cells, inhibiting Th1 proliferation [253]. Kaempferol, in HepG2 hepatoma cells, upregulates JAK1, TYK2, and STAT1/2, enhancing IFN-α-responsive gene expression and showing tumor-suppressive effects via interferon signaling [254]. ​Silibinin, known to inhibit STAT3 in lung and prostate cancers, also affects broader JAK/STAT pathways involving TYK2​ [255,256].

In summary, these findings suggest that flavonoids modulate TYK2-related JAK/STAT signaling in tumor and immune cells, offering potential to regulate cancer cell plasticity, including EMT, stemness, and resistance. Thus, flavonoids represent promising candidates for BC therapy targeting this pathway.

**Fig. 4:**
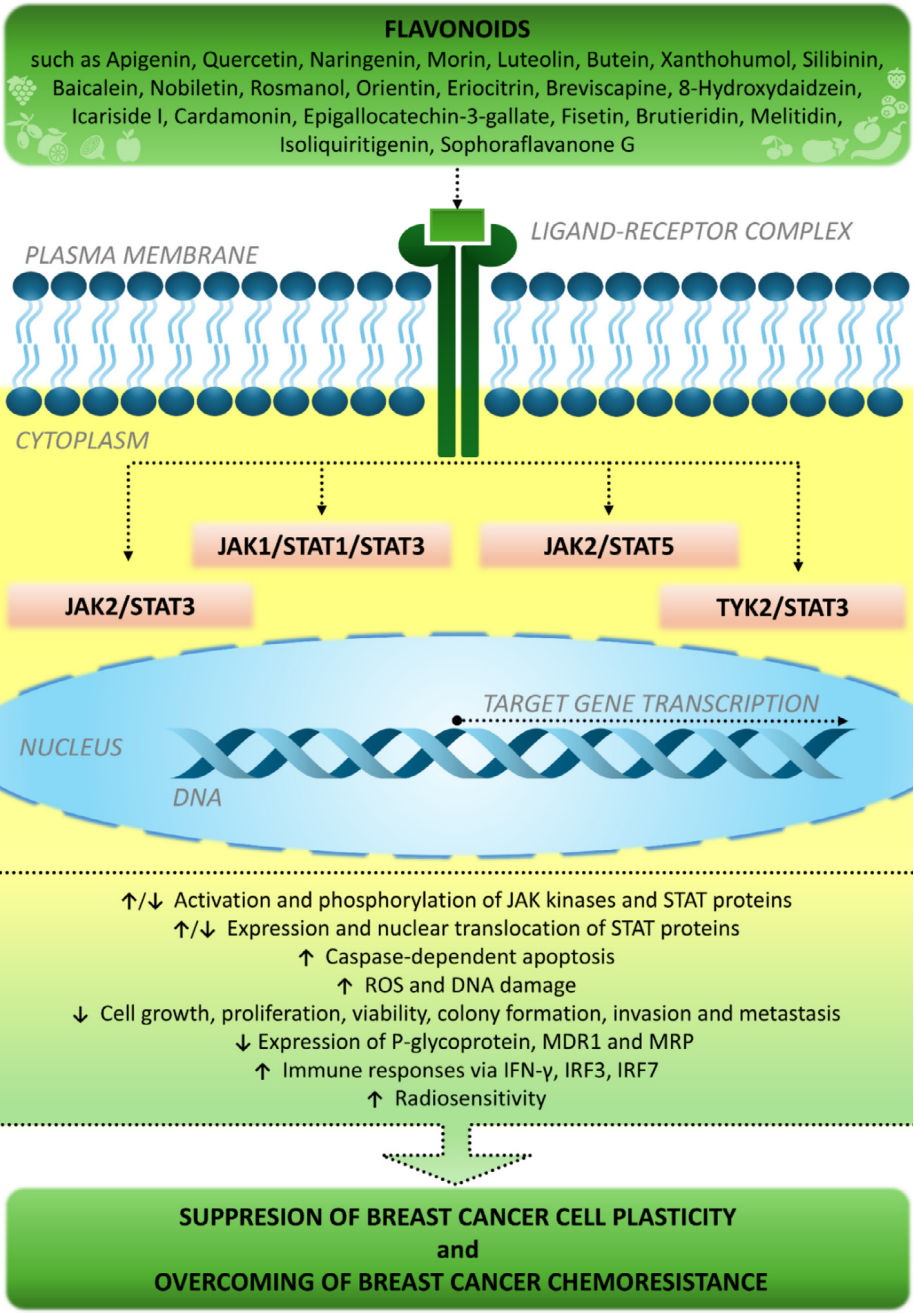
Targeting JAK/STAT Signaling with Flavonoids: A Multimodal Approach to Combat BC Resistance.

Fig. 4 illustrates the proposed mechanisms through which flavonoids modulate oncogenic JAK/STAT activity, reprogram tumor-immune interactions, and restore chemosensitivity in resistant BC phenotypes.

Dysregulated activation of Janus kinases (JAKs) within the JAK/STAT signaling cascade enhances the oncogenic potential of signal transducers and activators of transcription (STATs), thereby promoting BC cell survival and resistance to chemotherapy. Flavonoids, a class of polyphenolic compounds, have emerged as promising therapeutic agents in this context. Acting as potent tyrosine kinase inhibitors, flavonoids impede the activation and phosphorylation of both JAKs and STATs, subsequently diminishing STAT expression and nuclear translocation. This interference with JAK/STAT signaling attenuates key oncogenic processes, including cell proliferation, invasion, metastasis, and angiogenesis.

Moreover, flavonoids induce caspase-dependent apoptosis, DNA damage, and reactive oxygen species (ROS) generation. They also enhance the sensitivity of BC cells to chemotherapeutic and radiotherapeutic interventions while mitigating treatment-related toxicity. Certain flavonoids – such as apigenin, quercetin, naringenin, or morin – can activate the JAK/STAT pathway to stimulate antitumor immune responses. This immunomodulatory effect is mediated by regulating interferon-related signaling molecules, including interferon-gamma (IFN-γ), interferon regulatory factor 3 (IRF-3), and IRF-7.

**Abbreviations** **:**

↑ upregulation; ↓ downregulation; BC, breast cancer; IFN-γ, Interferon-gamma; IRF, Interferon regulatory factor; JAK, Janus kinase; MDR1 and MRP, Multidrug resistance proteins; ROS, Reactive oxygen species; STAT, signal transducer and activator of transcription protein; TYK2, Tyrosine kinase 2.

## Clinically relevant illustration of 3PM approach in primary and secondary care: AI-guided multiparametric analysis of JAK–STAT signaling

This section presents clinically relevant illustrations by analysing individual patient cases.

### Patient Case analysis 1: AI-guided risk stratification and targeted prevention of health-to-disease transition in primary care

A 31-year-old premenopausal woman presented with benign breast tissue alterations. To assess her predisposition to BC, a non-invasive, multi-parametric blood analysis was conducted, utilizing a specialized AI-based algorithm for pre-BC risk evaluation [47]. The analysis revealed proteomic signatures indicative of elevated BC risk, including increased levels of catalase and actin, a heightened Hcy-to-Comet Assay (CA) ratio, and the presence of hybridome CA-I/CA-IV patterns. Notably, reductions in CA-II, CA-III, and CA-IV patterns, as well as in the CA-I/CA-IV ratio, further supported classification of the patient as being at high risk for BC.

Based on these findings, personalized preventive strategies, including dietary and lifestyle interventions and regular clinical monitoring, were recommended to mitigate progression toward malignancy. An application of flavonoids as a dietary supplementation tailored to individualized patient profiles is recommended to support personalized protection against health-to-disease transition [257,258].

Relevance to JAK–STAT Signaling: Molecular features identified in this patient, including markers of mitochondrial dysfunction, chronic low-grade inflammation, and impaired tissue repair, are tightly associated with dysregulation of the JAK–STAT signaling pathway [[259], [260], [261]]. This pathway plays a pivotal role in the transition from benign to malignant phenotypes. As such, JAK–STAT signaling represents a promising therapeutic target for proactive intervention in high-risk individuals within primary care settings, particularly through strategies aimed at enhancing mitochondrial resilience and immune modulation [257,262,263].

### Patient case analysis 2: Phenotyping and individualized protection against disease progression in secondary care

A female premenopausal patient, 37 years old with a BMI = 21, was diagnosed with non-metastatic triple-negative BC. The patient is an evident Flammer syndrome phenotype (FSP) carrier demonstrating the phenotype-characteristic symptoms and signs including frequenly cold extremities and feeling cold in the environment where the temperature range is comfortable for others; typically pronounced pain sensitivity and slowed wound healing, dizziness, shifted circadian rhythms with prolonged sleep onset, altered reaction towards drugs as well as FSP-specific psychosomatic patterns (pronounced meticulous personality), amongst others.

The patient is concerned about:

• her mental, emotional, and physical health status suffering a lot due to the diagnosis and aggressive treatments that she underwent,
• her potential predisposition to metastatic disease later on in life.

Tear fluid analysis performed for the patient demonstrated blocked mitophagy compared to the reference values in the corresponding group of age, reflecting mitochondrial burnout (the know-how of 3PMedicon GmbH, Austria, performing internationally validated tests [264], with detailed protocols described elsewhere [265].

**Relevance of FSP and JAK–STAT Signaling to BC Pathogenesis and Progression:** The functional interrelationship between the FSP and JAK–STAT signaling is increasingly recognized as clinically relevant in BC, particularly in TNBC, influencing:

• **Circadian rhythm disruption**, linked to altered STAT signaling and tumor-promoting inflammatory pathways [[266], [267], [268], [269], [270]],
• Endothelin-1 upregulation, contributing to vasospasm, ischemia–reperfusion injury, and systemic stress responses [268,[271], [272], [273], [274]],
• Modified drug sensitivity, associated with altered expression of MDR transporters and JAK/STAT-mediated chemoresistance [266,268,[275], [276], [277], [278]],
• Delayed wound healing, further aggravated by mitochondrial dysfunction and systemic low-grade inflammation [258,271,[279], [280], [281], [282]].

**Therapeutic Implications:** A personalized rehabilitation plan was initiated, incorporating targeted interventions to restore mitochondrial function and systemic homeostasis. Strategies were tailored to the FSP phenotype and aimed at enhancing mitochondrial fitness and reducing the risk of disease progression [258,283]. Use flavonoids as dietary supplements aligned with individualized patient profiles to support personalized rehabilitation and help prevent disease progression [257,258].

## Concluding remarks and emerging avenues in flavonoid-based cancer therapy: From mechanistic insights to clinical applications

The JAK-STAT signaling pathway is integral to BC pathogenesis, influencing tumor progression, metastasis, and resistance to therapy. Flavonoids, a diverse group of naturally occurring polyphenolic compounds, have demonstrated the capacity to modulate this pathway, thereby affecting cancer cell plasticity and enhancing sensitivity to conventional treatments [26,38,187]. This positions flavonoids as promising agents within 3PM for BC management.

Persistent activation of the JAK-STAT pathway, particularly involving JAK2/STAT3 and JAK1/STAT3 axes, has been implicated in the development of chemoresistance, uncontrolled proliferation, and immune evasion in aggressive BC subtypes [65,86,111,116]. Flavonoids such as apigenin, quercetin, and naringenin have been shown to inhibit phosphorylation and activation of these signaling molecules, leading to reduced expression of downstream targets like MDR1, VEGF, and MMP-9, which are associated with drug resistance and metastasis. For instance, apigenin has been reported to downregulate MDR1 expression and inhibit JAK2/STAT3 activation in doxorubicin-resistant MCF-7 cells, thereby restoring drug sensitivity [44]. Similarly, quercetin has demonstrated the ability to suppress IL-6-induced JAK2/STAT3 signaling, enhancing the apoptotic response to chemotherapeutic agents in triple-negative BC models [42].

Cancer cell plasticity, characterized by the ability of cancer cells to undergo phenotypic changes such as EMT, contributes to therapy resistance and tumor progression. Flavonoids have been observed to interfere with signaling pathways that regulate plasticity [17,18]. For example, isoliquiritigenin has been shown to inhibit STAT3 signaling and downregulate miR-21 expression, a microRNA associated with EMT and stemness, in BC cells [202]. Moreover, the chalcone-syringaldehyde hybrid CSH1 has been reported to target focal adhesion kinase and STAT3 phosphorylation, thereby impeding cytoskeletal reorganization and invasive behavior in triple-negative BC cells [204].

Integrating flavonoid-induced modulation of the JAK-STAT signaling pathway into 3PM offers a promising strategy for optimizing BC management. By targeting cancer cell plasticity and mitigating drug resistance, flavonoids support individualized therapeutic approaches in line with 3PM principles [17,39,45]. Their anti-inflammatory and antioxidant properties, alongside the ability to inhibit EMT and CSC formation via JAK-STAT modulation, contribute to their chemopreventive potential [32,106,[284], [285], [286]]. Incorporating flavonoid profiles into predictive models may improve patient stratification and guide preventive strategies, particularly for high-risk individuals [36]. In therapeutic contexts, flavonoids can be co-administered with conventional agents to restore chemosensitivity and suppress tumor progression through synergistic mechanisms [39]. Realizing the full clinical utility of flavonoids requires a multidisciplinary research agenda focused on decoding their molecular interactions within JAK-STAT signaling, identifying predictive biomarkers, and developing advanced delivery platforms [287,288]. This integrative approach will enable the rational inclusion of flavonoids in precision oncology, ultimately enhancing therapeutic efficacy and personalizing care for BC patients.

### Future clinical directions

Advancing the clinical application of flavonoids necessitates a multifaceted research agenda that integrates several scientific approaches to support their effective translation into personalized BC therapies [34,289,290].

#### Clinical evaluation and mechanistic insights

While preclinical studies have demonstrated the potential of flavonoids to modulate the JAK-STAT signaling pathway and inhibit tumor progression, rigorous clinical trials are essential to validate their efficacy and safety in BC patients. Current research emphasizes their use in combination therapies rather than as standalone treatments to enhance the anticancer efficacy of flavonoids while minimizing systemic toxicity [291]. This approach leverages the synergistic effects of flavonoids with conventional chemotherapeutic agents, offering a more effective strategy for cancer management [291,292]. Additionally, in-depth mechanistic investigations are needed to elucidate the molecular interactions between flavonoids and components of the JAK-STAT pathway, which will inform the development of more potent and selective flavonoid derivatives.

#### Optimization of delivery systems

Encapsulation of flavonoids in biodegradable nanoparticles significantly enhances their anticancer efficacy while minimizing systemic toxicity by enabling targeted, in situ drug delivery to tumor sites [293,294]. The practical limitation of flavonoids is exposure (variable oral bioavailability), which is being addressed by nano-enabled and lipid-based formulations that improve systemic levels and sustain network engagement; this supports their rational use as partners with selective JAK/STAT inhibitors in resistance-prone settings [295,296].

Nanocarriers—such as polymeric nanoparticles, liposomes, and solid lipid nanoparticles—improve flavonoid solubility, stability, and enable sustained release for consistent therapeutic action [297,298].

Functionalization with ligands like antibodies or peptides further refines delivery specificity, minimizing off-target effects and enhancing treatment outcomes [299].

Overall, nanoparticle-based systems address the pharmacokinetic limitations of flavonoids and support their effective clinical application in cancer therapy [300].

#### Translational hurdles and solutions

**Standardization and analytics.** Adoption of best-practice guidelines for botanical products − chemical fingerprinting, quantitative marker panels, and transparent methods − is essential to control source/lot variability and enable cross-study comparability [190].

**Mechanism-anchored PK/PD and DDI profiling.** Early, mechanism-linked pharmacokinetic plans should quantify UGT/SULT conjugation and interrogate transporter interactions (P-gp, BCRP, MRPs) to anticipate off-target effects and drug–drug interactions, especially in combinations with kinase or immunotherapies [191].

**Formulation solutions.** Nano-enabled and lipid-based carriers (polymeric nanoparticles, liposomes, micelles, nanogels) improve solubility, stability, absorption, and target exposure for multiple flavonoids; pairing these with standardized source materials helps disentangle formulation from material variability [295].

**Clinical trial design.** Trials should (i) link standardized product chemistry to exposure (parent vs. conjugates), (ii) stratify by microbiome-relevant and metabolic covariates, and (iii) pre-specify interaction monitoring when co-administered with transporter- or enzyme-liability drugs [193].

**Net effect.** Addressing composition variability, limited selectivity/off-target risks, DDI potential, and formulation/exposure constraints provides a realistic path to move flavonoids from promise to practice in BC [301].

#### Biomarker discovery for patient stratification

Biomarkers of JAK/STAT pathway dysregulation are essential for predicting patient responsiveness to flavonoid-based therapies and guiding stratified treatment within the 3PM framework [31,285].

Advances in high-throughput genomic and proteomic platforms now enable comprehensive profiling of mutations, gene expression, and protein activity, facilitating the discovery of clinically relevant diagnostic and prognostic markers [302]. Specifically, elevated levels of phosphorylated STAT3 and STAT5A correlate with poor prognosis and treatment resistance in several cancers, including breast, colorectal, leukemia, and prostate [30].

Flavonoids such as apigenin and EGCG effectively inhibit JAK/STAT signaling by blocking protein phosphorylation, highlighting their therapeutic potential in pathway-specific interventions [303]. Leveraging integrated multi-omic analyses strengthens biomarker discovery and supports the personalized deployment of flavonoid-based therapies in oncology [304,305].

#### Synergistic potential of flavonoids in cancer therapy

Flavonoids like apigenin and quercetin can enhance the efficacy of chemotherapeutics by modulating key pathways (e.g., JAK/STAT, PI3K/Akt, MAPK, NF-kB), promoting apoptosis, and reducing proliferation [17,18,306]. They also help overcome chemoresistance by inhibiting drug efflux pumps and improving drug retention in tumor cells [307].

Combining flavonoids with agents like paclitaxel has shown synergistic effects, improving tumor control and reducing toxicity [308]. Continued research and clinical trials are needed to validate these combinations, particularly within personalized medicine frameworks [36,39,45].

#### Integration into the 3PM framework

The incorporation of flavonoid-based strategies into the 3PM paradigm offers a holistic approach to BC management [309]:

• **Predictive**: Clinicians can predict individual responses to flavonoid therapies by utilizing identified biomarkers and advanced patient phenotyping, allowing for proactive treatment planning.
• **Preventive**: Given their chemopreventive properties, flavonoids can reduce the risk of BC development, particularly in high-risk populations identified through predictive diagnostics.
• **Personalized**: By tailoring flavonoid-based treatments to patients' unique genetic and molecular profiles, personalized therapy regimens can be developed, enhancing efficacy and minimizing adverse effects.

#### Optimizing flavonoid-based JAK/STAT cancer therapies by application of artificial intelligence

Artificial intelligence (AI) is increasingly integral to the development of flavonoid-based therapies targeting the JAK/STAT signaling pathway in cancer treatment. Beyond biomarker discovery, AI contributes to multiple facets of therapy development, including clinical evaluation, optimization of delivery systems, design of synergistic combination therapies, and integration into the 3PM framework [310,311]. AI facilitates the analysis of complex clinical data, enabling the identification of patient subgroups that may benefit from flavonoid-based therapies. By integrating genomic, proteomic, and clinical data, AI models can predict treatment responses and elucidate mechanisms of action, enhancing our understanding of how flavonoids modulate the JAK/STAT pathway. This approach supports the development of personalized treatment strategies [312].

The therapeutic efficacy of flavonoids is often limited by poor bioavailability. AI-driven design and optimization of nanoparticle-based delivery systems can enhance the stability and targeted delivery of flavonoids to tumor sites. Machine learning algorithms assist in predicting optimal formulations, improving drug accumulation in tumors while minimizing systemic toxicity [313,314]. AI models are instrumental in identifying effective combinations of flavonoids with existing chemotherapeutic agents. By analyzing vast datasets, AI can predict synergistic interactions that enhance anticancer efficacy and overcome drug resistance [[315], [316], [317], [318]].

This accelerates the discovery of combination therapies that are more effective than monotherapies. AI supports the 3PM approach by enabling predictive analytics, patient stratification, and personalized therapy design. Through the analysis of multi-omics data, AI aids in identifying individuals at high risk of cancer development and tailoring preventive strategies accordingly [319]. This integration ensures that flavonoid-based therapies are aligned with the principles of 3PM.

In conclusion, a concerted effort encompassing clinical validation, mechanistic research, delivery optimization, biomarker discovery, and integration into the 3PM framework is imperative to fully realize flavonoids' potential in BC therapy. Such an approach promises to advance personalized oncology care and improve patient outcomes.

## Declarations

### Data availability

No datasets were generated or analyzed during the current study.

## Code availability

Not applicable.

## Clinical trial number

Not applicable.

## Compliance with ethics Requirements

All patients included in the study were informed about the purposes of the study and had signed their “consent of the patient”. All investigations conformed to the principles outlined in the Declaration of Helsinki and were performed with corresponding permissions (Nr. 148/05 and 283/10) released by the responsible ethics committee of the Medical Faculty, Rheinische Friedrich-Wilhelms-University of Bonn.

## Ethics approval

All authors have read and agreed to the published version of the manuscript. All patients included in the study were informed about the purposes of the study and had signed their “consent of the patient”. All investigations conformed to the principles outlined in the Declaration of Helsinki and were performed with corresponding permissions (Nr. 148/05 and 283/10) released by the responsible ethics committee of the Medical Faculty, Rheinische Friedrich-Wilhelms-10.13039/501100008131University of Bonn.

The graphical abstract included in this article was generated using OpenAI's ChatGPT with image generation capabilities, based on the authors' original scientific content and data. The grammar and style of this manuscript were checked using Grammarly software, version 1.2.166.1677.

## Funding

Open Access funding is enabled and organized by Project DEAL. The present study was supported by the European Association for Predictive, Preventive and Personalised Medicine, EPMA, Brussels, and by the 10.13039/501100006109Scientific Grant Agency of the 10.13039/501100003193Ministry of Education, Science, Research and Sport of the Slovak Republic (Bratislava, Slovak Republic; grant no. 10.13039/501100006109VEGA 1/0045/23, VEGA 1/0122/24, VEGA 1/0236/23, VEGA 1/0145/22, and VEGA 1/0286/22).

## Declaration of competing interests

The authors declare no competing interests.
